# Long noncoding RNA intersectin 1-2 gradually declines during adalimumab treatment, and its reduction correlates with treatment efficacy in patients with ankylosing spondylitis

**DOI:** 10.1007/s10787-021-00854-3

**Published:** 2021-08-18

**Authors:** Mingwu Li, Xianjie Zhou

**Affiliations:** Edong Healthcare Group, Department of Orthopedics, Huangshi Central Hospital, Affiliated Hospital of Hubei Polytechnic University, 141 Tianjin Road, Huangshi Port District, Huangshi, 435000 China

**Keywords:** Lnc-ITSN1-2, Ankylosing spondylitis, Disease risk, TNFα inhibitors, Treatment efficacy

## Abstract

Previous studies show that long noncoding RNA intersectin 1-2 (lnc-ITSN1-2) promotes the inflammation process and serves as a potential biomarker in autoimmune diseases, except for ankylosing spondylitis (AS). Therefore, this study aimed to explore the correlation of baseline lnc-ITSN1-2 expression with disease risk and activity of AS, and to investigate its longitudinal change with treatment response to a tumour necrosis factor alpha (TNFα) inhibitor in patients with AS. In total, 63 patients with AS receiving 12-week adalimumab treatment were included and their baseline clinical features were collected. Lnc-ITSN1-2 expression in peripheral blood mononuclear cells (PBMC) of patients with AS was detected by reverse transcription quantitative polymerase chain reaction. Meanwhile, Assessment in Spondyloarthritis International Society (ASAS) 40 response was evaluated at week 2 (W2), W4, W8, and W12. According to the ASAS40 response status at W12, patients with AS were classified into responders and non-responders. PBMC lnc-ITSN1-2 expression was also determined in healthy controls (*N* = 60). Lnc-ITSN1-2 expression was elevated in patients with AS compared to controls (*P* < 0.001). Baseline lnc-ITSN1-2 expression was positively associated with C-reaction protein (CRP) (*P* = 0.021), interleukin (IL)-1β (*P* = 0.020), Bath Ankylosing Spondylitis Disease Activity Index (BASDAI) score (*P* = 0.040), and Ankylosing Spondylitis Disease Activity score with C-reactive protein (ASDAS_CRP_) (*P* = 0.045) in patients with AS. Furthermore, lnc-ITSN1-2 expression declined during the treatment with adalimumab (*P* < 0.001). Notably, this reduction was more obvious in responders than non-responders. In conclusion, declined lnc-ITSN1-2 expression during the TNFα inhibitor treatment correlates with good treatment efficacy in patients with AS, suggesting its clinical value for AS management.

## Introduction

Ankylosing spondylitis (AS) is a chronic inflammatory disease that mainly affects the axial skeleton with unknown etiology (Smith 2015). Regarding its management, medications (including nonsteroidal anti-inflammatory drugs, short-term glucocorticoids, and biologics) are commonly prescribed to remit pain and reduce inflammation in patients with AS (Sari et al. 2015). Tumor necrosis factor alpha (TNFα) inhibitors (such as adalimumab, etanercept, infliximab, and golimumab) are commonly used biologics that can achieve rapid reduction of inflammation in patients with AS and further display promising clinical efficacy with a high safety profile following their approval for AS management (Corbett et al. 2016; Maxwell et al. 2015). However, some patients with AS may experience low response to TNFα inhibitors which decreases their long-term prognosis (Terenzi et al. 2018). Therefore, finding novel and reliable biomarkers to predict treatment efficacy of TNFα inhibitors in patients with AS is needed.

Long noncoding RNAs (lncRNAs) are a collection of noncoding RNAs of relatively large size involved in numerous cellular and biological processes (Akhade et al. 2017). As a newly identified lncRNA, lncRNA intersectin 1-2 (lnc-ITSN1-2) may induce inflammation in the fibroblast-like synoviocytes (FLS) of rheumatoid arthritis (RA) through promoting the nucleotide-binding oligomerization domain 2 (NOD2)/receptor-interacting protein 2 (RIP2) axis (Yue et al. 2019). Moreover, lnc-ITSN1-2 may promote the differentiation of T helper type 1 (Th1) and Th17 cells in CD4^+^ T cells of inflammatory bowel disease (IBD) via regulating the microRNA (miR)-125a/interleukin (IL)-23R axis (Nie and Zhao 2020). In the clinical field, lnc-ITSN1-2 expression correlates with RA risk and IBD risk, and it correlates with elevated disease activity in patients with RA and patients with IBD (Gong et al. 2017; Nie and Zhao 2020; Yue et al. 2019). Furthermore, lnc-ITSN1-2 expression is reduced after the TNFα inhibitor treatment in patients with active IBD (Nie and Zhao 2020). However, no relevant study has been reported regarding the clinical application of lnc-ITSN1-2 for AS management. Therefore, we conducted this study aiming to explore the correlation of lnc-ITSN1-2 expression with disease risk and activity of AS. More importantly, we also intended to investigate lnc-ITSN1-2 longitudinal change during the TNFα inhibitor (adalimumab) treatment and its association with treatment efficacy in patients with AS.

## Materials and methods

### Patients

This study was permitted by the institutional review board, and all subjects signed informed consent documents. This prospective cohort study enrolled 63 patients with AS between January 2019 and July 2020. The recruitment criteria were as follows: (i) diagnosis of AS; (ii) aged older than 18 years; (iii) had active disease which was defined as Bath Ankylosing Spondylitis Disease Activity Index (BASDAI) > 4 (based on a 10-cm visual analogue scale, VAS) and Ankylosing Spondylitis Disease Activity Score with C-reactive protein (ASDAS_CRP_) > 2.1; (iv) willing to receive a 12-week adalimumab treatment. The patients were ineligible for inclusion if they met any of the following exclusion criteria: (i) known contraindications to adalimumab; (ii) dysfunction in liver, kidney, heart, or respiration; (iii) active infection; (iv) complicated with malignancies; (v) poor compliance to the study. Additionally, 60 healthy subjects who underwent physical examination in our hospital were recruited as healthy controls. In order to allow matching of the age and gender between healthy controls and the patients with AS, the age of healthy controls was limited to within 18–50 years, and the gender of healthy controls was limited at a ratio of 4:1 (male vs. female).

### Evaluation of baseline characteristics

Demographics, medical history, and inflammatory biomarkers of patients with AS were recorded after clinical and laboratory examinations. The following indicators were evaluated to assess baseline disease activity which included BASDAI, Bath Ankylosing Spondylitis Functional Index (BASFI), total back pain, Patient’s Global Assessment of Disease Activity (PGADA), and ASDAS_CRP_. The evaluation of the BASDAI, BASFI, total back pain, and PGADA was performed using a 10-cm VAS.

### Sample collection and determination

Peripheral blood (PB) samples from patients with AS were collected prior to starting treatment (W0) to determine the level of inflammatory cytokines including TNFα, interleukin (IL)-1β, IL-6, and IL-17A. Serum was separated from the PB samples in a refrigerated centrifuge by centrifuging at 1000 to 2000×*g* for 10 min and then used for determining the level of inflammatory cytokines by enzyme-linked immunosorbent assay (ELISA) using commercial human ELISA kits (Shanghai Enzyme-linked Biotechnology Co., Ltd, Shanghai, China). For measurement of lnc-ITSN1-2 expression during the adalimumab treatment, PB samples of patients with AS were also collected at week 2 (W2), week 4 (W4), week 8 (W8), and week 12 (W12) after initiation of treatment, except for W0. Immediately after each sample collection, peripheral blood mononuclear cells (PBMC) were isolated by the density gradient separation method and then used to determine the lnc-ITSN1-2 expression by reverse transcription quantitative polymerase chain reaction (RT-qPCR). As for healthy controls, PB samples were obtained during their physical examination and then were used for separation of PBMC to determine the lnc-ITSN1-2 expression by RT-qPCR as described below.

### RT-qPCR assay

The lnc-ITSN1-2 expression from PBMC was detected by RT-qPCR. In brief, TRIzol™ Reagent (Invitrogen, Waltham, Massachusetts, USA) was used to isolate total RNA. After extraction, iScript™ cDNA Synthesis Kit (with random primer) (Bio-Rad, Hercules, California, USA) was used for cDNA synthesis. Then the PCR reaction was completed using QuantiNova SYBR Green PCR Kit (QiaGen, Duesseldorf, Nordrhein-Westfalen, Germany). The lnc-ITSN1-2 relative expression was calculated by the 2^−ΔΔCt^ method with GAPDH as an internal reference. The primer sequence was designed according to a previous study as follows: lnc-ITSN1-2, forward primer (5′–3′) GCTTCACTCGCTTGCTTACA; reverse primer (5′–3′) GGTTCTGTCTTGCCTTCTGTT; GAPDH, forward primer (5′–3′) GACCACAGTCCATGCCATCAC; reverse primer (5′–3′) ACGCCTGCTTCACCACCTT (Livak and Schmittgen 2001).

### Treatment with adalimumab and assessment

All patients received a 12-week adalimumab treatment by receiving 40 mg adalimumab (Abbott Laboratories, Chicago, Illinois, USA) through subcutaneous injection every 2 weeks. During the treatment, the treatment response was assessed at W2, W4, W8, and W12 using Assessment in Spondyloarthritis International Society (ASAS) 40 criteria and the detailed definition of the ASAS40 response was described in previous literature (Sieper et al. 2009). For study analysis, subjects were categorized as responders if they had an ASAS40 response at W12; otherwise they were categorized as non-responders.

### Statistical analysis

Comparison of lnc-ITSN1-2 expression between two groups was examined by Mann–Whitney *U* test. Repeated measures of lnc-ITSN1-2 expression among different time points were analyzed using the Friedman test. Correlation of lnc-ITSN1-2 expression with other variables was analyzed by Spearman’s correlation test. Receiver operating characteristic (ROC) curve analysis was used to determine the performance of lnc-ITSN1-2 expression in distinguishing different subjects. A *P* value less than 0.05 was considered as statistical significance. SPSS 21.0 (IBM Corp., Armonk, New York, USA) was employed to execute data analysis.

## Results

### Patients’ baseline features

The mean age of patients with AS was 34.0 ± 8.3 years (Table 1). The number of female and male patients was 9 (14.3%) and 54 (85.7%), respectively. The median disease duration of patients with AS was 6.0 (4.0–9.0) years. There were 16 (25.4%) patients who had a history of TNFα inhibitor treatment. Other clinical information regarding disease activity, biochemical indexes, and inflammatory cytokine levels in patients with AS are listed in Table 1.

**Table 1:** Baseline characteristics of patients with AS

Items	Patients with AS (*N* = 63)
Demographic characteristics	
Age (years), mean ± SD	34.0 ± 8.3
Gender, no. (%)	
Female	9 (14.3)
Male	54 (85.7)
Disease characteristics	
Disease duration (years), median (IQR)	6.0 (4.0–9.0)
0–5 years, no. (%)	26 (41.3)
6–10 years, no. (%)	29 (46.0)
> 10 years, no. (%)	8 (12.7)
History of TNFα inhibitor, no. (%)	
Yes	16 (25.4)
No	47 (74.6)
HLA-B27 positive, no. (%)	
Yes	57 (90.5)
No	6 (9.5)
Biochemical indexes, median (IQR)	
CRP (mg/L)	28.1 (19.1–38.0)
ESR (mm/h)	31.9 (21.6–43.9)
Disease activity scores, mean ± SD	
BASDAI score	6.3 ± 1.0
BASFI score	4.8 ± 1.1
Total back pain score	6.7 ± 1.3
PGADA score	6.6 ± 1.4
ASDAS_CRP_	4.0 ± 0.8
Inflammatory cytokines, median (IQR)	
TNFα (pg/mL)	55.3 (42.1–78.8)
IL-1β (pg/mL)	5.8 (4.1–7.9)
IL-6 (pg/mL)	38.9 (29.5–54.6)
IL-17A (pg/mL)	105.5 (57.5–123.7)

*AS* ankylosing spondylitis, *SD* standard deviation, *IQR* interquartile range, *TNF* tumor necrosis factor, *HLA-B27* human leukocyte antigen B27, *CRP* C-reactive protein, *ESR* erythrocyte sedimentation rate, *BASDAI* Bath Ankylosing Spondylitis Disease Activity Index, *BASFI* Bath Ankylosing Spondylitis Functional Index, *PGADA* Patient Global Assessment of Disease Activity, *ASDAS*_*CRP*_ Ankylosing Spondylitis Disease Activity Score with C-reactive protein, *IL* interleukin

### Comparison of lnc-ITSN1-2 expression between patients with AS and controls

**Fig. 1:**
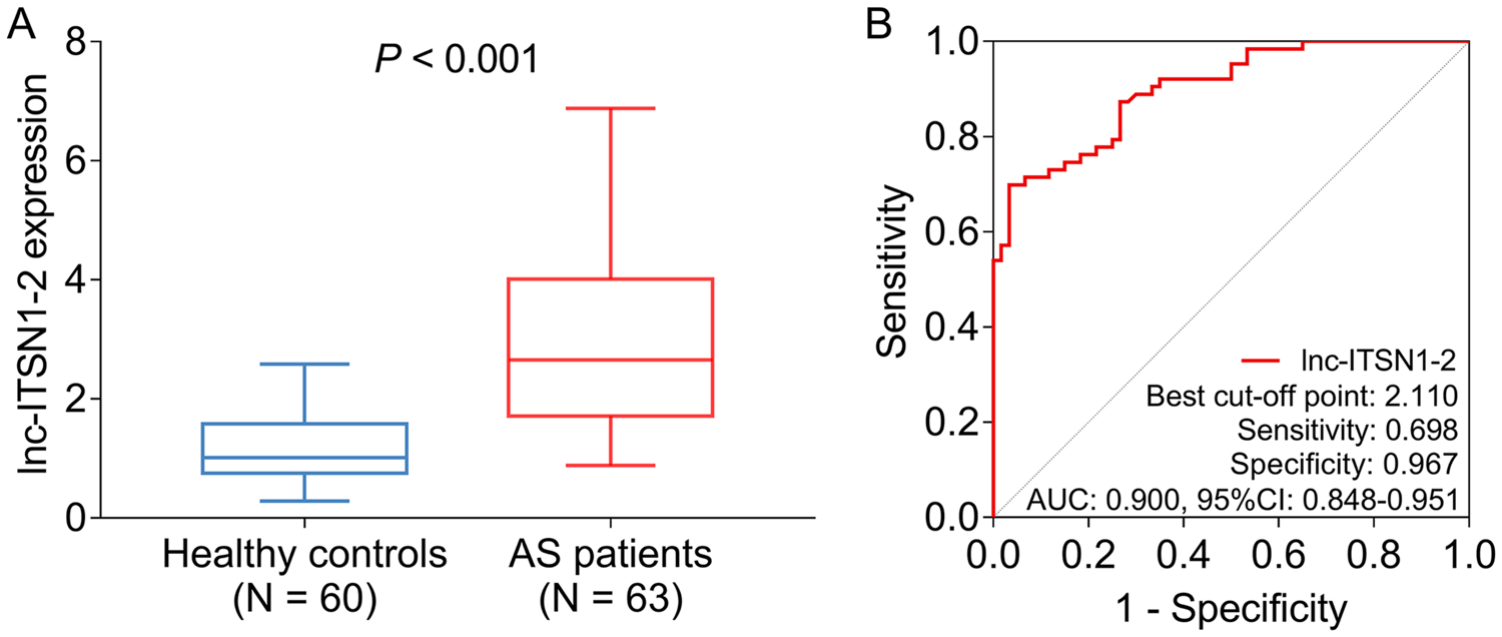
Lnc-ITSN1-2 expression correlated with AS risk. Lnc-ITSN1-2 expression was increased in patients with AS compared to controls (**a**), and it could differentiate patients with AS from controls (**b**) by ROC curve analysis. Lnc-ITSN1-2 long noncoding RNA intersectin 1-2, AS ankylosing spondylitis, ROC receiver operating characteristic, AUC area under curve, CI confidence interval

Lnc-ITSN1-2 expression was elevated in patients with AS compared to controls (*P* < 0.001) (Fig. 1a). The ROC curve showed that lnc-ITSN1-2 expression had excellent potential in discriminating patients with AS from healthy controls with an AUC of 0.900 (95% CI 0.848–0.951); its specificity and specificity of the best cutoff point were 0.967 and 0.698, respectively (Fig. 1b).

### Correlation of baseline lnc-ITSN1-2 expression with clinical features and inflammatory cytokines in patients with AS

**Fig. 2:**
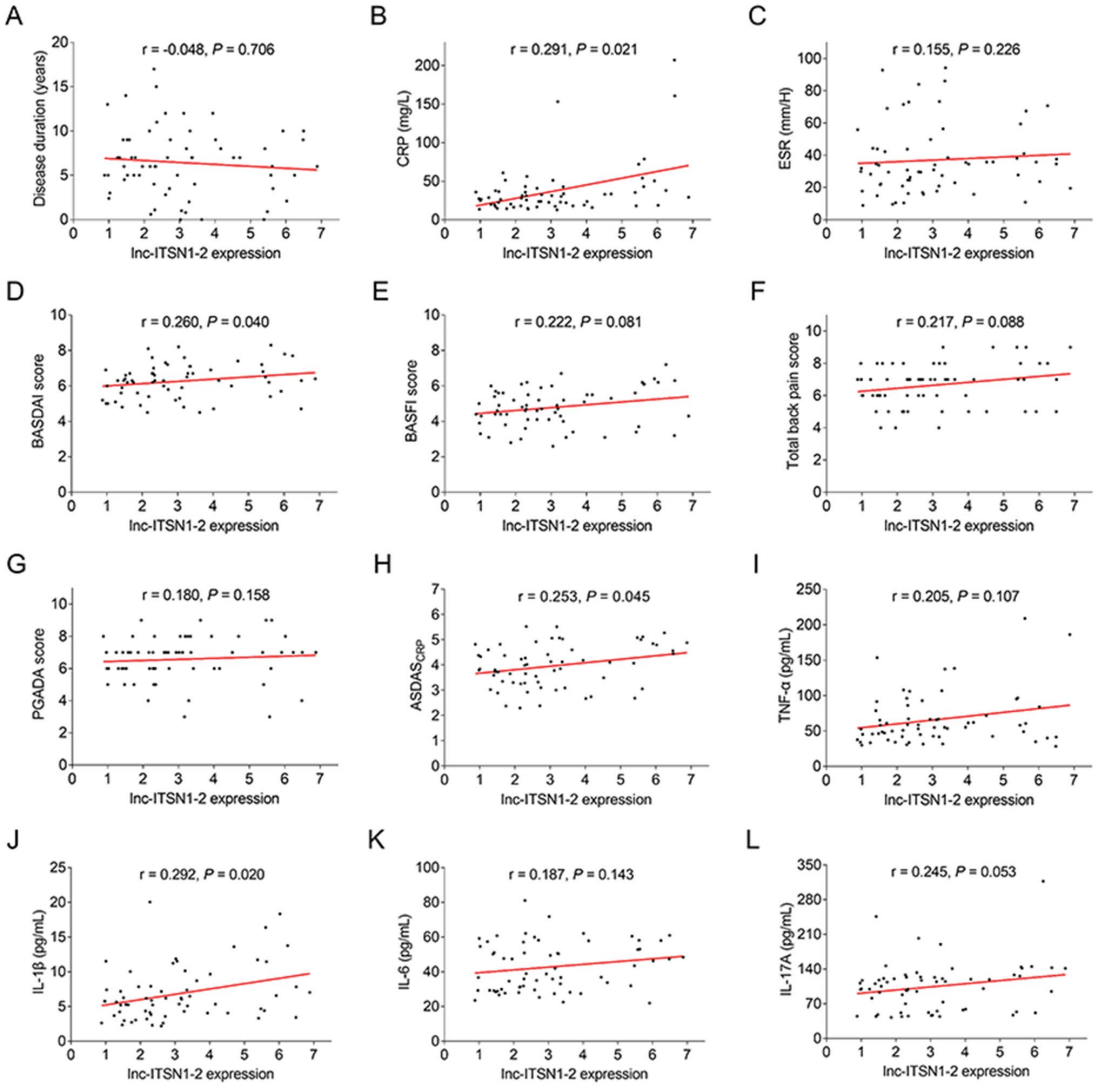
Lnc-ITSN1-2 expression positively correlated with inflammation and disease activity in patients with AS. The correlation of lnc-ITSN1-2 expression with disease duration (**A**), CRP (**B**), ESR (**C**), BASDAI score (**D**), BASFI score (**E**), total back pain score (**F**), PGADA score (**G**), ASDAS_CRP_ (**H**) TNFα (**I**), IL-1β (**J**), IL-6 (**K**), and IL-17A (**L**) in patients with AS. Lnc-ITSN1-2 long noncoding RNA intersectin 1-2, AS ankylosing spondylitis, CRP C-reaction protein, ESR erythrocyte sedimentation rate, BASDAI Bath Ankylosing Spondylitis Disease Activity Index, BASFI Bath Ankylosing Spondylitis Functional Index, PGADA Patient’s Global Assessment of Disease Activity, ASDAS_CRP_ Ankylosing Spondylitis Disease Activity Score with C-reactive protein, TNF tumor necrosis factor, IL interleukin

Lnc-ITSN1-2 expression positively associated with CRP (*r* = 0.291, *P* = 0.021) (Fig. 2b), BASDAI score (*r* = 0.260, *P* = 0.040) (Fig. 2d), ASDAS_CRP_ (*r* = 0.253, *P* = 0.045) (Fig. 2h), and IL-1β (*r* = 0.292, *P* = 0.020) (Fig. 2j) in patients with AS. However, the lnc-ITSN1-2 expression was not related to disease duration (*r* = − 0.048, *P* = 0.706) (Fig. 2a), ESR (*r* = 0.155, *P* = 0.226) (Fig. 2c), BASFI score (*r* = 0.222, *P* = 0.081) (Fig. 2e), total back pain score (*r* = 0.217, *P* = 0.088) (Fig. 2f), PGADA score (*r* = 0.108, *P* = 0.158) (Fig. 2g), TNFα (*r* = 0.205, *P* = 0.107) (Fig. 2i), IL-6 (*r* = 0.187, *P* = 0.143) (Fig. 2k), or IL-17A (*r* = 0.245, *P* = 0.053) (Fig. 2l) in patients with AS.

### Longitudinal change of lnc-ITSN1-2 expression during treatment in patients with AS

**Fig. 3:**
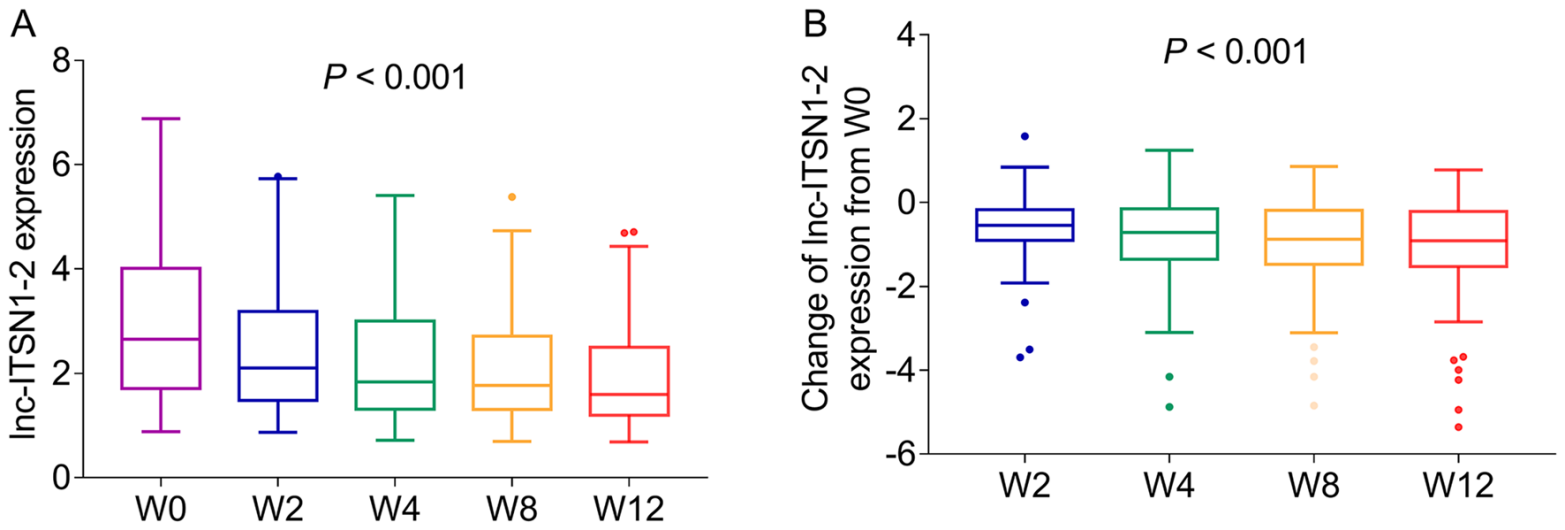
Lnc-ITSN1-2 expression was reduced during adalimumab treatment in patients with AS. Longitudinal change of lnc-ITSN1-2 expression (**A**) and the change of lnc-ITSN1-2 expression from baseline to different time points (**B**) during adalimumab treatment in patients with AS. Lnc-ITSN1-2 long noncoding RNA intersectin 1-2, AS ankylosing spondylitis, W week

During treatment, lnc-ITSN1-2 expression was decreased from W0 to W12 in patients with AS (*P* < 0.001) (Fig. 3a). Additionally, a reduction of lnc-ITSN1-2 expression was also observed from W2 to W12 in patients with AS (*P* < 0.001) (Fig. 3b).

### Treatment response in patients with AS

**Fig. 4:**
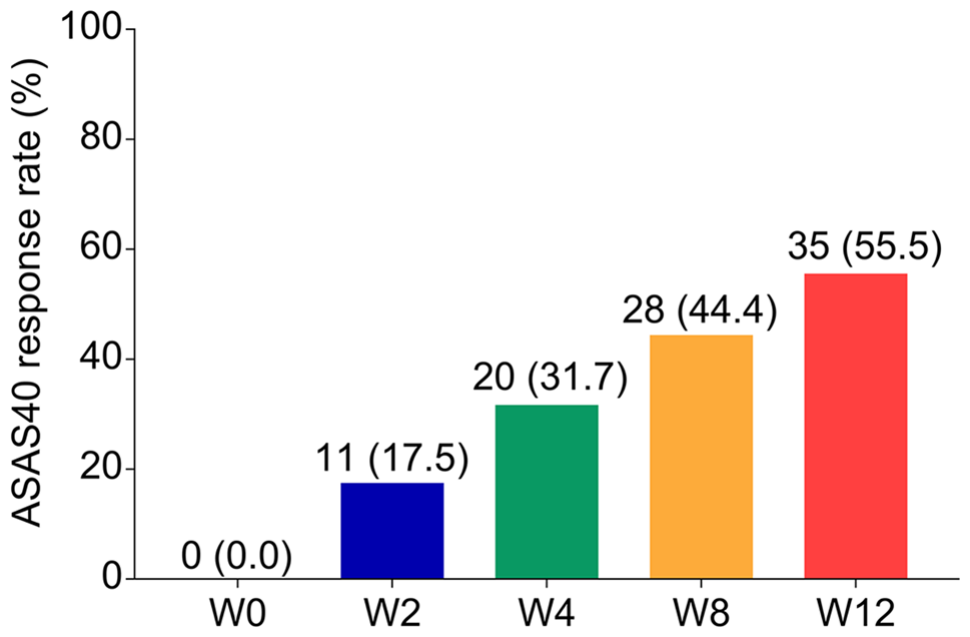
ASAS40 response rate at different time points. ASAS Assessment in Spondyloarthritis International Society, W week

The evaluation of ASAS40 response was conducted at each time point during treatment in patients with AS. There were 0 (0.0%), 11 (17.5%), 20 (31.7%), 28 (44.4%), and 35 (55.5%) patients who achieved ASAS40 response at W0, W2, W4, W8, and W12, respectively (Fig. 4).

### Correlation of lnc-ITSN1-2 longitudinal change with treatment response in patients with AS

**Fig. 5:**
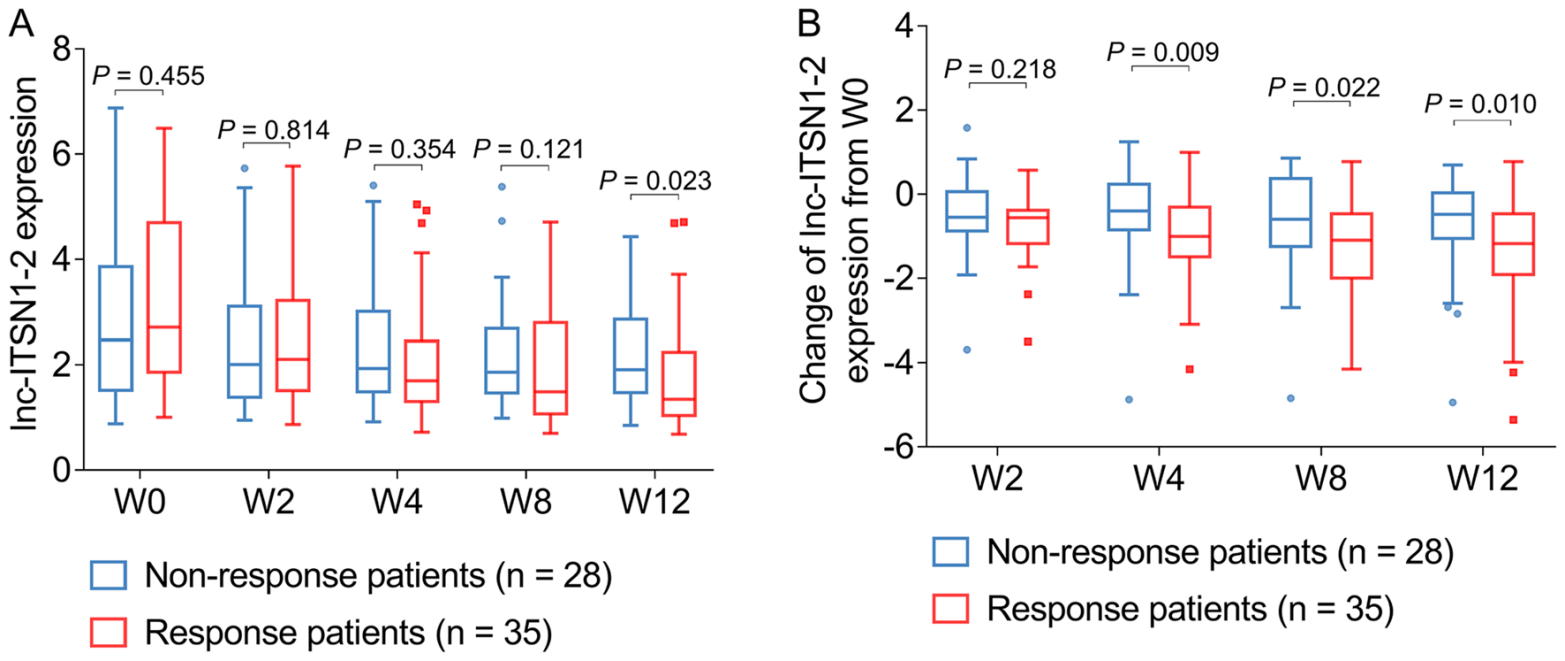
Lnc-ITSN1-2 expression was reduced in responders compared to non-responders. Comparison of lnc-ITSN1-2 expression (**A**) and change of lnc-ITSN1-2 expression from W0 (**B**) at different time points between responders with AS and non-responders with AS. Lnc-ITSN1-2 long noncoding RNA intersectin 1-2, AS ankylosing spondylitis, W week

Lnc-ITSN1-2 expression at W12 was reduced in responders compared to non-responders (*P* = 0.023) (Fig. 5a). Furthermore, the change of lnc-ITSN1-2 expression from W0 was also decreased in responders compared to non-responders at W4 (*P* = 0.009), at W8 (*P* = 0.022) and at W12 (*P* = 0.010) (Fig. 5b).

**Fig. 6:**
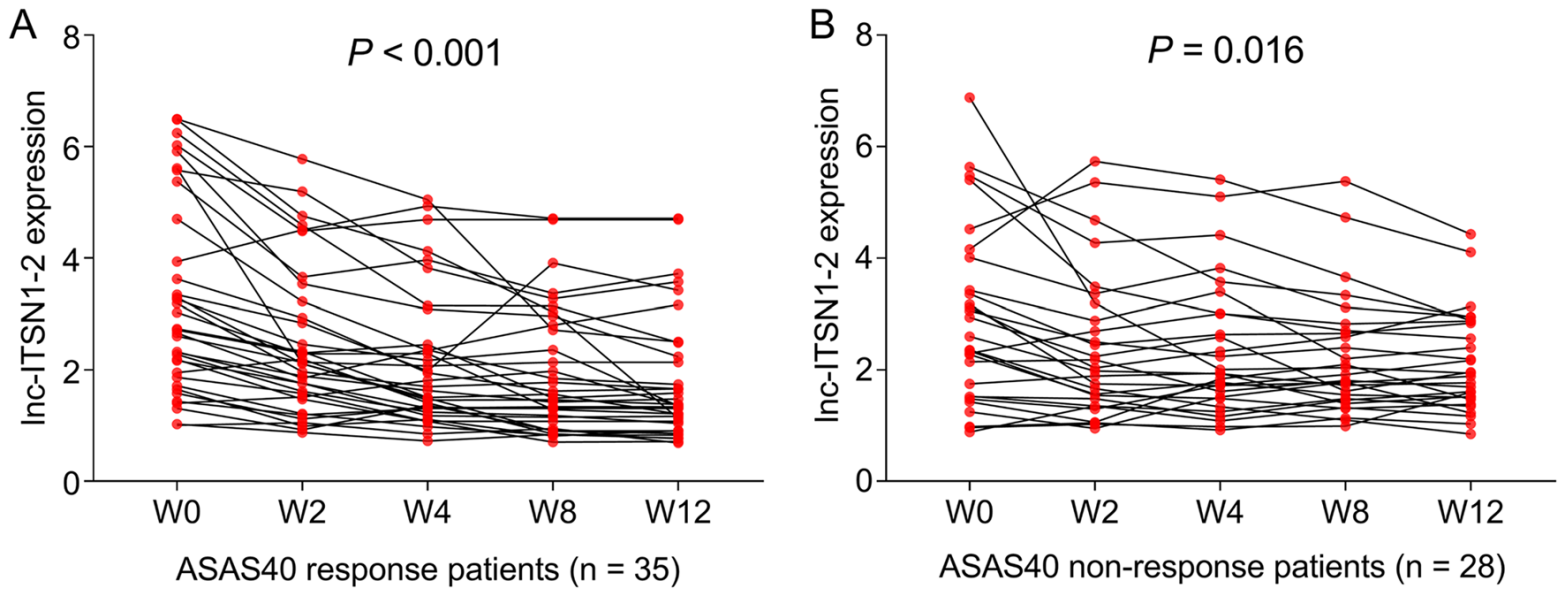
Reduction of lnc-ITSN1-2 expression during adalimumab treatment was more obvious in responders with AS. Longitudinal change of lnc-ITSN1-2 expression in ASAS40 responders (**A**) and in ASAS40 non-responders with AS (**B**). Lnc-ITSN1-2 long noncoding RNA intersectin 1-2, AS ankylosing spondylitis, W week, ASAS Assessment in Spondyloarthritis International Society

In addition, an obvious declination of lnc-ITSN1-2 expression was observed from W0 to W12 in ASAS40 responders (*P* < 0.001) (Fig. 6a). A similar tendency was also observed in ASAS40 non-responders (*P* = 0.016) (Fig. 6b). Interestingly, a more obvious reduction of lnc-ITSN1-2 expression during treatment was observed in ASAS40 responders.

## Discussion

Lnc-ITSN1-2 is a recently identified lncRNA which might play a critical role in inflammation-mediated diseases. Previous clinical studies show that lnc-ITSN1-2 expression is upregulated and its high expression correlates with elevated inflammation (including CRP, TNFα, and IL-17A) and increased disease activity reflected by DAS28 score in patients with RA (Gong et al. 2017; Yue et al. 2019). lnc-ITSN1-2 expression also correlates with disease risk and active disease status of IBD, and it positively correlates with CRP, ESR, and Crohn’s disease activity index in patients with active Crohn’s disease (CD) (Nie and Zhao 2020). However, no relevant study has reported on the clinical role of lnc-ITSN1-2 in patients with AS. Therefore, we performed this study and discovered that lnc-ITSN1-2 expression was elevated in patients with AS compared to controls and it also correlated with AS risk. Additionally, baseline lnc-ITSN1-2 expression was positively related to inflammation (CRP and IL-1β) and disease activity (BASDAI score and ASDAS_CRP_) in patients with AS. Possible reasons might be that (a) Lnc-ITSN1-2 might promote the production of inflammatory cytokines (such as TNFα) and lead to increased inflammation and osteoclastogenesis (osteoclast formation), which further causes an imbalance between bone formation and bone destruction. Therefore, lnc-ITSN1-2 expression correlated with elevated AS risk (Cici et al. 2019; Yue et al. 2019); (b) Lnc-ITSN1-2 promoted CD4^+^ T cell activation, proliferation, and Th1/Th17 cell differentiation though regulating the miR-125a/IL-23R axis, then led to enhanced immune response and higher inflammation which eventually result in elevated AS risk (Nie and Zhao 2020); (c) Upregulation of lnc-ITSN1-2 expression was related to higher inflammatory cytokine production (such as TNFα, IL-17, and IFNγ), which led to an elevated inflammatory condition and a higher disease activity in patients with AS (Corbett et al. 2016; Maxwell et al. 2015; Yue et al. 2019).

As commonly used biologics, TNFα inhibitors reduce inflammation rapidly through restraining TNFα binding to its receptor and further suppressing downstream inflammatory cytokine production (Corbett et al. 2016; Maxwell et al. 2015). After promising efficacy was achieved with less safety concerns in several large-scale randomized controlled trials (RCTs), the TNFα inhibitor (adalimumab) was approved for AS (Sieper et al. 2013; van der Heijde et al. 2006). However, some problems still existed, such as poor treatment response in some patients and emerging presence of anti-drug antibodies to TNFα inhibitors (Gorovits et al. 2018; Terenzi et al. 2018). Thus, identifying potential biomarkers to improve treatment response and clinical outcomes in patients with AS is necessary. A previous study discovered that lnc-ITSN1-2 expression is decreased after the TNFα inhibitor treatment in patients with active CD, suggesting it may serve as a potential biomarker to predict treatment efficacy of TNFα inhibitor (Nie and Zhao 2020). In the present study, we found that lnc-ITSN1-2 expression was reduced during the TNFα inhibitor treatment, and its reduction was related to treatment efficacy of the TNFα inhibitor in patients with AS. These findings might be explained as follows: (a) reduced lnc-ITSN1-2 expression correlated with less inflammation as discussed earlier, which suggested a treatment response to adalimumab (Gong et al. 2017; Nie and Zhao 2020; Yue et al. 2019); (b) as from the clinical experience, responders might achieve a great reduction of inflammation and disease activity compared to non-responders. Therefore, the reduction of lnc-ITSN1-2 expression during the TNFα inhibitor treatment might be more obvious in responders and further associated with treatment efficacy in patients with AS.

Several limitations existed in this study. Firstly, the sample size was relatively small and therefore further study with a larger sample size is needed to validate these findings. Secondly, the underlying mechanism of lnc-ITSN1-2 expression in AS was not investigated; thus, it might be necessary to conduct relevant molecular studies. Finally, our study only investigated the correlation of longitudinal change of lnc-ITSN1-2 expression with treatment efficacy of adalimumab in patients with AS, while its association with treatment efficacy of other types of TNFα inhibitors, such as etanercept or infliximab, was not investigated. Hence, further study could be performed.

In conclusion, reduced lnc-ITSN1-2 expression during the TNFα inhibitor treatment correlates with treatment efficacy in patients with AS, suggesting its clinical value for AS management.
